# The Impact of Nurse Practitioners on Hospitalizations and Discharges from Long-term Nursing Facilities: A Systematic Review

**DOI:** 10.3390/healthcare8020114

**Published:** 2020-04-28

**Authors:** Michael Mileski, Upwinder Pannu, Bobbi Payne, Erica Sterling, Rebecca McClay

**Affiliations:** 1 School of Health Administration, Texas State University, San Marcos, TX 78666, USA; 2 School of Science, Technology, Engineering, and Mathematics, American Public University System, Charles Town, WV 25414, USA

**Keywords:** nurse practitioner, hospitalization, long-term care, skilled nursing facility, nursing home, resident care, quality of care, quality outcomes

## Abstract

The objective of this study was to increase the understanding of the role a nurse practitioner (NP) has in reducing the risk of hospitalizations and improving quality outcomes among nursing facility residents. This was explored by the research team conducting a systematic literature review via Cumulative Index of Nursing and Allied Health Literature, PubMed (MEDLINE), and Academic Search Ultimate. This is of concern because of the increased rate of hospital readmissions from skilled nursing facilities. The study found that utilization of NPs as primary care providers resulted in decreased unnecessary hospitalizations, increased access to healthcare, and improved health outcomes. NPs are fundamental in building relationships with residents and families and providing them information for decision making. The utilization of NPs in a long-term care setting should be encouraged to improve access to care, decrease hospitalizations, and enhance quality of care. States with reduced or restrictive scope of practice for NPs should revisit the regulations to provide unrestricted scope of practice for NPs.

## 1. Introduction

Nurse Practitioners (NPs) are trained to provide primary care in multiple settings including adult, family, pediatric heath, women’s health, and gerontology. NPs play a variety of roles in the long-term care setting to include providing both acute and primary care to short-term and long-term care residents, staff consultation on patient issues, and education to residents, families, and staff [1]. Primary services provided by NPs have been found to provide equivalent or more effective chronic disease management in treatment of hypertension, diabetes, depression, and congestive heart failure, than physicians [1,2]. Improved outcomes were noted in functional status of residents with NPs as primary care providers [1]. Nursing facilities with NPs were found to have lower hospitalization rates for diagnoses such as congestive heart failure, chronic obstructive pulmonary disease, hypertension, asthma, and diabetes [1,3,4]. The odds of ambulatory care sensitive hospitalization were 43% lower and 50% lower in Alzheimer’s disease or dementia-related diagnoses in facilities with an onsite NP [1,4,5]. Decreased hospitalization results in reducing the cost of care [1,4]. NPs provided a timelier response, spent more time with residents, and conducted more comprehensive assessments than physicians [1]. Onsite NPs improved staff morale, resident satisfaction, and family satisfaction [1,4,5].

### 1.1. Background

Nursing homes are a vital element of the long-term care system. They play an important role in caring for the most sick, frail, and vulnerable elderly adults. The United States Census Bureau has projected that by 2050, the number of Americans aged 85 years and older will be approximately 19 million [2]. Nursing facilities provide services to meet the medical and psychosocial needs of residents. They also are expected to provide high standards for quality of care and quality of life. Quality of care and quality of life include effectively managing transitional care. Transitional care is coordinating continuity of care to other healthcare facilities or back to the community [3]. The transfer of a resident to a hospital is an example of transitional care.

Nursing home residents are at higher risk for hospitalizations [3,4]. One-fifth of all Medicare beneficiaries were reported to be readmitted to the hospital within 30 days of discharge [3]. It is estimated that 90% of these hospitalizations were unplanned and cost Medicare approximately $17.4 billion [4]. Many of the rehospitalizations were considered to be preventable such as rehospitalizations secondary to congestive heart failure (CHF), respiratory infections, urinary tract infections (UTI), sepsis, and electrolyte imbalance [4,5]. Hospitalizations due to the five aforementioned conditions accounted for 78% of potentially avoidable thirty-day skilled nursing facility rehospitalizations [4]. It is estimated that potentially avoidable rehospitalizations from a skilled nursing facility cost Medicare $3.39 billion in 2004 [4]. It is reported that one in six nursing home residents have a hospital admission within any given six-month period [5]. Approximately 40% of the long-term care residents have a hospital transfer within 30 days prior to their deaths [5]. Some of the causes of preventable hospital readmissions include inadequate management of chronic conditions, inadequate management of infections, and other unplanned events [1,4].

### 1.2. Significance

The diagnostic-related group (DRGs) payment system in hospitals has resulted in shorter hospital length of stays [1,3,4]. It has resulted in hospitals discharging patients “quicker and sicker” to nursing facilities [1]. The increase in the number of nursing home residents with clinically complex needs poses a challenge for facilities and physicians to meet them [1,4]. The addition of NPs to the clinical staff can help meet resident needs, provide quality of care, and reduce hospitalizations [1].

The main objective of this literature review is to investigate the role of an NP in reducing hospitalizations and improving quality outcomes in long-term care settings. The study will identify both areas for successes and barriers with the role of the NP.

## 2. Materials and Methods

### 2.1. Design

This study used a systematic review of peer-reviewed articles found in three research databases, Academic Search Ultimate (ASU), CINAHL Complete, and PubMed. The benchmark of acceptability for the topic and Boolean search was no less than 30 unduplicated articles from the three research databases. Using a 4-string Boolean phrase, each author searched for articles pertaining to the research question. This study used terms outside normal MeSH terms, as we were able to realize a much richer base of articles when we utilized different terms for our Boolean search. The initial search was conducted on 22 September 2019. Upon reviewing the articles found during the initial search, the authors used cited articles from the reference lists which led to additional articles meeting the inclusion criteria. The final articles were found on 19 October 2019, completing the search for data needed for the systematic review. The Preferred Reporting Items for Systematic Reviews (PRISMA) guidelines were used to ensure consistent and precise reporting of search results. This review was also constructed and conceptualized using the Kruse Protocol for writing systematic reviews [6].

### 2.2. Inclusion Criteria

All authors individually reviewed the articles from the searches. Articles were eligible for inclusion if they were published by academic journals or universities between 1 January 2004 and 30 August 2019. The articles had to be published in the English language and pertaining to humans only. Articles had to explore nurse practitioners impacting hospital admissions or readmissions for patients in long-term care to be included in the systematic review. 

### 2.3. Exclusion Criteria

Articles were only incorporated if deemed germane by all authors. Articles that were systematic reviews, literature reviews, or metanalyses were excluded. Articles that pertained to nurse practitioners in acute care were not included. Analysis of projected proposed use of nurse practitioners or conceptual models were excluded. The review did not include comparisons of physicians to nurse practitioners in relation to hospital readmissions. Bias was not considered when reviewing the research involved in this study. The final sample of articles after meeting exclusion criteria was then analyzed further for consensus among all authors for final inclusion. When analyzed, the sample yielded a kappa statistic (k = 1), showing strong reliability.

## 3. Results

### 3.1. Study Selection

**Figure 1:**
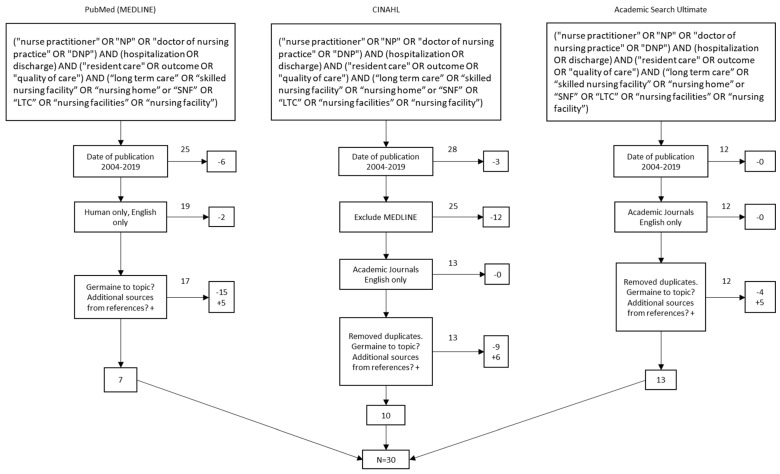
Preferred Reporting Items for Systematic Reviews Flow Diagram (PRISMA).

The article selection process is outlined in the PRISMA flow diagram in Figure 1. The initial search protocol identified a total of 65 articles from the three databases. Eleven articles were excluded when articles were filtered to include English and humans only, published between 1 January 2004 and 31 December 2019. Researchers chose to extend the search parameters to fifteen years due to a lack of relevant research in the field. Forty-two articles remained and 28 duplicates articles were removed leaving a total of 14 articles. Upon reviewing the articles found during the initial search, the authors used cited articles from the references lists which led to additional 16 germane articles. The total number of articles for the systematic review was 30.

### 3.2. Assessment Tools

The 30 articles selected for the systematic review were summarized to include the author, aim, setting, methods, assessment tool, and key findings. The summary of articles is listed in Table 1. The authors examined and analyzed the 30 selected articles, identified facilitator and barrier themes, and then sorted the articles according to their respective facilitator or barrier theme. Using the Affinity Matrix, facilitator and barrier themes associated with NPs reducing hospitalizations and improving quality outcomes in long-term care settings were listed by frequency of occurrences using articles numbers. The sum and percentages of the frequencies of facilitator and barrier themes were then calculated. The Affinity Matrix diagram is demonstrated in Table 2.

## 4. Results

A total of 30 articles published over a fifteen-year period, 2004 through 2019, were reviewed. All the articles reviewed discussed the impact nurse practitioners had in reducing hospitalizations and improving quality outcomes in the long-term care setting. Themes were identified and sorted into positive facilitators and negative barriers impacting nurse practitioners and patient care with regard to hospital admissions or readmission to hospitals for residents of long-term care facilities. Nineteen themes were identified, ten of which were facilitators, nine of which were barriers. 

### 4.1. Facilitators 

Ten facilitator themes were identified. Their occurrence, frequency sum, and percent frequency are shown in Table 2. The positive facilitator theme presence of NPs decreasing hospitalizations was mentioned in 37 of 136 occurrences, or 27.21% [7,8,9,10,11,12,13,14,15,16,17,18,19,20,21,22,23,24,25,26,27]. The presence of NPs also improved health outcomes was mentioned in 27 of 136 occurrences of facilitator themes, or 19.85% [7,9,12,14,20,21,23,24,26,28,29,30,31]. Improved patient and family satisfaction were mentioned in 2 of 136 occurrences of facilitator themes, or 1.47% [19,32]. Improved quality of care was mentioned in 26 of 136 occurrences of facilitator themes, or 19.12% [8,13,14,18,19,20,23,24,26,27,28,31,33]. Improved end-of-life care was mentioned in 3 of 136 occurrences of facilitator themes, or 2.21% [16]. Additional facilitator themes identified during the review of the articles were as follows. Increased access to healthcare was mentioned in 14 of 136 occurrences of facilitator themes, or 10.29% [18,20,23,24,25,30,32,33,34]. Patient and family education were mentioned in 3 of 136 occurrences of facilitator themes, 2.21% [13,20]. Multidisciplinary approach was mentioned in 5 of 136 occurrences of facilitator themes, or 3.68% [21,25,29,32]. Reduced healthcare costs were mentioned in 9 of 136 occurrences of facilitator themes, or 6.62% [7,8,9,12,14,23,30]. Unrestrictive or least restrictive scope of practice for NPs was mentioned in 10 of 136 occurrences of facilitator themes, 7.35% [10,14,16,17,31,32,33,34,35,36].

Successful strategies in which NPs decreased hospitalizations included NP-led advance care planning, medication reconciliation interventions, employing full-time NP at skilled nursing facilities, NPs providing patient-centered care, increased access and availability of full-practice NPs, staff coaching and education, and on-site assessments [7,8,10,11,12,13,17,20,22,23,24,25,26].

Many of the articles reviewed indicated NPs were a valuable resource to prevent unnecessary hospitalizations and clearly impacted a reduction in emergency room visits and hospitalizations particularly among the frail elderly and seniors [7,8,9,10,11,12,13,14,15,16,17,18,20,21,22,23,24,25,26,27]. Elderly patients managed by NPs had a lower hospital length of stay, were provided person-centered care, and a significant difference was found in the relationship between the number of NP discussions with patients about care and the number of hospitalizations [7,20,23]. Skilled nursing facilities implementing an NP-led medication reconciliation intervention lead to reduction in all-cause hospital readmissions [8]. 

Improved health outcomes were associated with NP interventions and care, as indicated in 19.85% of occurrences [7,8,9,12,13,14,16,17,18,19,20,21,23,24,27,28,29,30,32]. NPs, as primary care providers, advocated for patients and families, improved chronic disease management and care planning, and reduced depressive symptoms in patients they treated [20,24,29]. Time NPs spent in nursing activities were highly correlated with improved health outcomes, as well [9,24,28]. For example, when NPs provided only two hours on average per month for six months, elders in their care were able to improve function with a hip fracture and increase their activities of daily living [24]. Patients transferring between inpatient hospitals and nursing facilities had improved outcomes when a full-time NP was on staff [28]. Additionally, patients seen by NPs within four days of admission to a long-term care facility had lower mortality and costs during the following month [9]. Once residents and their families experienced NP care, patient satisfaction and family and patient requests for NP services improved [19,32].

Improved quality of care was mentioned in 23 of 132 occurrences (19.21%) of facilitator themes encompassing the improvement of quality measures, providing education to staff and assisting with clinical care, and involvement with quality improvement initiatives, e.g., the Intervention to Reduce Acute Care Transfers (INTERACT) [7,8,9,12,13,14,16,17,18,19,20,21,23,24,27,28,29,30,32,33]. One qualitative study reviewed provided insights to person-centered care from the residents’ and family perspectives [23]. Relationships between the patients, families, and the NP were fundamental to the experience of life within the community of a long-term care home [23]. Not only was the quality of care improved by having the NPs work in these settings, but also both residents and family members expressed how they valued NP’s sharing information with them and involving them in decision-making related to care [23].

When NPs were utilized, patients experienced increased access to healthcare, patient and family education, and a multidisciplinary approach [13,18,20,21,23,24,25,29,30,32,33]. Increased access to healthcare was mentioned in 14 of 132 occurrences (10.29%) of facilitator themes [18,20,23,24,25,30,32,33,34]. Failures in the traditional process of primary care provider referrals negatively impacted quality outcomes of resident care [32]. When utilized, NP services adequately filled the gap [32]. In fact, in long-term care, NPs often played the lead role as primary care providers [34]. Their ability to communicate and collaborate with other healthcare professionals, patients, and their families about their chronic illnesses, disease trajectories, and their goals of care positively impacted clinical outcomes [20,21]. Additionally, NPs provided staff education to address underlying causes of falls and other medical conditions such as dehydration, muscle weakness, and medication side effects further improving clinical outcomes [13].

The theme of reduced healthcare costs was mentioned in 9 of 132 factors of facilitation (6.62%). NPs reduced healthcare costs, lowering Medicare expenditures per patient by reducing emergency room visits and providing cost-effective care [7,8,9,11,12,14,23,30]. One study reviewed indicated that when integrating NPs to care, patient savings of $13,000 were seen when hospital charges were compared between the cohorts [7]. Additionally, NPs increased revenue by keeping the patients within the long-term care setting for treatment [8].

The theme NPs working in an unrestrictive or least restrictive scope of practice occurred in 10 of 136 occurrences (7.35%). Those NPs working at this capacity were well positioned to promote high quality care, multidisciplinary collaboration, communication, education, leadership, advocacy, research, and evidence-informed practice [10,14,16,17,31,33,34,36]. Unrestricted scope of practice for NPs improved health outcomes and reduced the costs [34]. To ensure successful NP practice, both state and organizational policies should be taken into consideration [36].

NPs positively impacted end-of-life care for residents in nursing home facilities [16]. Themes of improving end-of-life care were mentioned in 2.21% of the occurrences. With the advent of NPs with palliative care expertise, end-of-life nursing home care was improved by reducing acute care use and potentially burdensome care transitions for residents with advanced illness [16]. 

### 4.2. Barriers

Nine barrier themes were identified. Their occurrence, frequency sum, and percent frequency are shown in Table 2. The negative barrier theme most often mentioned was the restrictive scope of practice for NPs, identified in 13 of 45 occurrences (28.89%) reviewed [9,14,16,20,23,24,31,32,34,35,36]. Lack of policy and funding, Medicare regulations, state regulatory constraints, prohibited billing for visits, and no authority to write or change orders were all contributors to restrictive scope of practice for NPs [9,23,24,32,34,35]. These restrictions required direct or indirect supervision from physicians for NPs, as well [31]. Changes in Medicare regulations are needed to facilitate improved utilization of NP services in long term care facilities [14].

Poor quality of care was identified as another barrier in 11 of 45 occurrences (24.44%) [13,19,20,22,27,28]. In some cases, the use of NPs as primary providers caused delays in care of the resident and outcomes, however, these outcomes were not significant when compared to physician outcomes [28]. Poor discharge protocols from hospitals to nursing facilities caused issues with rehospitalization before residents could be seen by NPs [13,28]. Further issues with discharge and admission to nursing facilities caused approximately 27.9% of residents to not be seen by physicians or NPs before hospital readmission [28]. This contributed to increased mortality rates of residents due to slowed time from admission to treatment by NPs [19]. Many facilities used objective tools at admission to assess acuity of residents and these tools were not used appropriately, thus increasing time to NP intervention [20]. There was also some evidence of increased mortality rates [19] and a lack of improvement of the care being given by NPs [27], however these were not more significant than care provided by physicians.

Lack of access to healthcare was identified as another barrier to the use of NPs in 7 of 45 occurrences (15.56%) [17,20,22,23,27,29]. Unfortunately, choices made by families or residents often resulted in discharges to hospital instead of care being provided by NPs [29]. Conversely, there was also a lack of available NPs in many areas to provide care to residents [17]. Both this lack of available staff and lack of specifically trained NPs in behavioral interventions resulted in a lack of proper behavioral health care being provided [17,20]. Nursing facility residents have a 25% greater chance at acquiring chronic conditions [22]. Of these chronic conditions, there are five that account for approximately 80% of hospital admissions/readmissions which are difficult for any provider to manage [22]. There is a perception that NPs are not aggressive enough in encouraging rehabilitation [27], however it should be noted that this is relatively dictated by resident payor status to begin with. When NPs were tasked with covering too many nursing facilities, this caused a decrease in the level of care [23].

Inadequate staffing was also identified as a concern surrounding the use of NPs in 6 of 45 occurrences (13.33%) [8,10,17,23,28,33]. Many long-term care facilities operated with limited resources and inadequate staffing further increasing the barriers NPs faced [8,10,17,23,28,33]. Many long-term care facilities do not have the capacity or resources to have an available NP to perform a stabilization and medication reconciliation visit upon each admission, for example [8]. Additionally, many transfers occurred on evening and night shifts which had lower staffing levels [17].

Patient and family preferences due to decreased patient satisfaction were also identified, occurring in 6.67% of the occurrences. Both were identified as barriers to NPs’ impact to reduce hospitalizations and improve quality outcomes in the long-term care setting [17,23,29]. Residents may prefer to get treatment and/or insist to go to the hospital, for example, instead of receive care from the NP at the long-term care facility [17,23]. It is probable that in circumstances where acute illness or injury arise, the preference for the transfer of patients predominates even when alternatives exist [29].

Poor decision making [18*] (4.44%), increased hospitalizations [19] (2.22%), increased healthcare costs [19] (2.22%), and poor communication [36] (2.22%) were also identified as barriers. Poor practices by the discharging hospitals resulting in early rehospitalizations occurred many times before patients could be evaluated by an NP [19]. For those patients admitted to the long-term facility, many were transferred back to the hospital within 30 days of admission due to functional decline, suspected respiratory infections, and new urinary tract infections [18]. Hospitalizations from these conditions could have been potentially avoided should an NP have provided care and treatment [18]. For those communities utilizing NP services, NPs often covered a high number of residents over multiple homes negatively affecting the quality of care and increasing patient transfers to hospitals [36]. Management changes, i.e., administrators, directors of nursing, and other key personnel may also negatively impact the already existing deficiencies in NPs’ perceptions of their relationship with administration and how their role is understood and valued in their organizations [18,19].

## 5. Discussion

The study findings indicate that NPs play an important role in improving health outcomes, quality of care, and reducing hospitalizations in a long-term care setting. The facilitators of NP roles outweigh the barriers in the long-term care setting. State quality initiatives show clear benefits of the use of full-time advanced practice registered nurses. These benefits are in quality improvement activities, increased end-of-life decision making, and improvements in the use of health information technology. Benefits of the use of systematic care tracking tools such as the use of the Interventions to Reduce Acute Care Transfers (INTERACT) were also noted. Avoidable hospitalizations actually increased under the use of NPs by 7% overall, however unavoidable hospitalizations decreased by an impressive 17%. The use of NPs has allowed a much more directed examination of care delivery systems, and measures to be put into place to correct any deficiencies in this area. The use of NPs to review medications and to reconcile them resulted in a 5.7% decrease in unnecessary hospitalizations.

Overall, the use of NPs reduces acute care usage, hospitalizations, and it eases care transitions when they do occur. NPs bring an increased ability to build relationships with families and residents, and to help both populations to better understand the care and to make well-informed choices. These are all significant areas which contribute to an increased quality of care which occurs when a NP is involved. There is a marked difference in health outcomes and hospitalizations in states with high levels of NP involvement as opposed to states with lower involvement. The utilization of NPs in a long-term care setting should be encouraged to improve access to care and to enhance quality of care.

## 6. Limitations

The authors identified limitations to this review. Our search strategy relied on a 4-string Boolean phrase and germane topics assigned by authors and may have missed instruments that are relevant to the role of an NP in reducing hospitalizations and improving quality outcomes in long-term care settings but were not identified. Many of the articles reviewed had small sample sizes which limited the generalizability of the individual study results. Additionally, because each long-term care facility had different dynamics, the results of this review may not be generalizable. Many long-term care facilities do not have the capacity or resources to have an available NP. Only English language articles were included in the review, so we did not capture the perspectives of people from different backgrounds.

### Future Research

An unexpected finding during the systematic review was the restrictive state and federal regulations regarding NPs. Future research and review of the different restrictions could assist with enabling NPs to practice more freely, improving quality outcomes and decreasing hospitalizations. More research using larger samples in the area of NP impact on long-term care quality outcomes and decreasing hospitalizations would increase the generalizability of study results. Future studies should address NPs’ impact on decreased hospitalizations and improved quality outcomes other than those in primarily English-speaking regions. Further areas of future research may also include the impact of NPs on staffing shortages surrounding physician providers. The inclusion of NPs could also significantly change the administration of medical care in nursing facilities. However, the role of the NP could also prove to be challenging as well when it comes to the provision of that care and the differences in states with full practice rights for NPs and those without.

## Figures and Tables

**Table 1:** Studies selected for review and a summary of design, methods, and results.

Author Last Name	Aim	Sample/Settings	Method	Assessment Tool	Key Findings
Popejoy	Exploring the differences in potentially avoidable/unavoidable hospital transfers in a retrospective analysis of INTERACT (Interventions to Reduce Acute Care Transfers, ACTs (Acute Transfer Tools) completed by advanced practice registered nurses (APRNs) working in the Missouri Quality Improvement Initiative (MOQI)	16 nursing homes, ranging from 120 to 321 beds	Cross-sectional descriptive study	INTERACT and ACT	Over one-half of the transfers (54%) were identified as avoidable transfers. Clinical factors related to avoidable transfers included falls, fever, urinary symptoms/incontinence. The transfers had a condition which could have been managed in a nursing home (58%), transfers could have been avoided with better communication (39%), new signs/symptoms detected earlier (31%), and advance directives in place earlier (11%)
Ouslander, Naharci	To determine the types of SNF to hospital transfers that occur within 48 h and 30 days of SNF admission based on root cause analyses (RCAs) and to identify potential areas for improving transitional care between hospitals and SNFs	88 skilled nursing facilities	Trained staff from SNFs enrolled in a randomized, controlled clinical trial of the INTERACT (Interventions to Reduce Acute Care Transfers) quality improvement program performed retrospective RCAs on hospital transfers during a 12-month implementation period	INTERACT (Interventions to Reduce Acute Care Transfers) Quality Improvement (QI) tool, retrospective RCA (root cause analyses) on hospital transfers	First, more intensive monitoring of these patients during the first 48 h to 7 days after SNF admission may help identify changes in condition early enough to intervene before hospital transfer is necessary. Teams of physicians and nurse practitioners have been shown to be effective in reducing hospitalizations and potentially avoidable hospitalizations in particular
Bethea	To compare the outcomes in elderly patients whose care was coordinated by trauma nurse practitioner (TNP) versus non trauma NP (NTNP)	1363 patients were analyzed for this study in a Level 1 trauma center between December 2014 and June 2017	Retrospective cohort study	Patient demographics, comorbidities, admission injuries, Injury Severity Score (ISS), hospital length of stay and unplanned ICU admission. Study outcomes also based on discharge location including home, skilled nursing, in-hospital mortality, 30-day readmission and hospital charges	Shorter length of stay for patients under care of TNPs which resulted in decreased hospital charges of $13,000 per admission. Patient under care of TNPs demonstrated higher percentage of home discharges as compared to discharge to skilled nursing facilities
Rantz, Popejoy	To determine if the Missouri Quality Initiative (MOQI) for long-stay nursing home residents reduced the frequency of preventable hospitalizations, improved resident health outcomes, improved the process of transition of care between hospitals and nursing facilities and reduced healthcare costs	16 nursing homes in metro, urban and rural communities	Prospective, single group intervention design	Interventions to Reduce Acute Care Transfers (INTERACT), INTERACT RCA (Root Cause Analysis), Early illness identification (stop and watch), SBAR (Situation, background, assessment, recommendation)	The MOQI (Missouri Quality Initiative) achieved 30% reduction in all-cause hospitalizations with full-time APRNs working in each facility and supported by the MOQI team to assist with quality improvement activities, consistent use of INTERACT, increased end-of-life decision making, and improved use of HIT (health information technology) for secure communication. There was a decline in the nonavoidable transfers from 64% to 47% and an increase in the percentage of avoidable from 47% to 54%
Rantz, Birtley	To study the implementation of an inter-professional model in nursing facilities with advanced practice registered nurses (APRNs) with aim to reduce preventable hospitalizations among nursing home residents	16 nursing homes. SNF, LTC	Retrospective, quasi-experimental	Physician and APRN reimbursement by CPT codes. Allowable APRN visits and orders for SNF versus NF	The model results demonstrated significant reduction in hospitalizations (40% all cause, 58% potentially avoidable), emergency room visits (54% all cause, 65% potentially avoidable), Medicare expenditures for hospitalizations (34% all cause, 45% potentially avoidable), and Medicare expenditures for emergency room visits (50% all cause, 60% potentially avoidable)
Ersek	To reduce the burden of costly hospitalizations of nursing home residents by using the Transforming Institutional Care Project (OPTIMISTIC)	19 skilled nursing facilities. Participants included 23 nursing home staff and leaders, 4 primary care providers, 10 family members, and 26 OPTIMISTIC clinical staff	One time, semi structured qualitative interviews	Advance care planning (ACP), Transforming Institutional Care (OPTIMISTIC), INTERACT	This project provided NPs time to facilitate high quality ACP discussions, conduct comprehensive resident assessment, timely identification and management of acute changes, staff education, thorough discussions with residents and families regarding care plan goals and analysis of root causes for potentially preventable hospitalizations
Yang	To determine which clinical specialties are most significant to care of individuals with dementia in the community and long-term care (LTC) settings	Long term care setting and Community setting. Fee-for-service Medicare beneficiaries with dementia aged 65 years or older	Cross-sectional analysis	Patient characteristics were examined by specialty of the PPC (predominant provider of care) and by community versus LTC settings using t test or chi-squared tests, as appropriate. The maps were used to show geographic variation at the state level for NPs as the PPC of care	More than 90% of beneficiaries had primary care providers as their PPC in LTC compared with 77.3% of beneficiaries in the community. Among the primary care providers, NPs as PPCs had the greatest difference between community (6.9%) and LTC (19.2%). NPs play as PPCs in LTC and how their role differs across states. In LTC, 40 states had NPs serving as PPCs for 12% to 40% of dementia beneficiaries living in LTC facilities
Meunier	The research determines if the use of comprehensive models, such as Program of All-Inclusive Care for the Elderly (PACE), lead to improved functional outcomes for participants and cost savings through decreased utilization	Long term care in community setting. 34 former participants in PACE were monitored every 6 months for 2 years and data was collected	Retrospective, quasi-experimental	Physical Self-Maintenance Scale, Instrumental Activities of Daily Living Scale, 6-point Likert response scale, Saint Louis University Mental Status Exam (SLUMS)	Both number of ED visits and hospitalizations were found to be significantly higher after PACE closure. For every hour of home health per month, the number of ED/hospital visits decreased in a 6-month period by 5.4%. Over the 2-year study period, functional scores ADLs and IADLs significantly declined. The majority of participants (67%) reported a higher level of satisfaction with PACE services as compared to usual care provided post-PACE
Oliver	To examine the relationship between the level of advanced practice registered nurse (APRN) practice (full, reduced, or restricted) allowed and results of analyses of Medicare or Medicare-Medicaid beneficiaries of possibly preventable hospitalizations, readmission rates after inpatient rehabilitation, and nursing home resident hospitalizations	Hospitals, Inpatient rehab, SNF and LTC	Retrospective	Two-sampled t-tests, One-way analysis of variance, Tukey test	States with the highest level of scope of practice for NPs showed reduced hospitalizations and enhanced health outcomes. The study indicates that any type of barriers which restrict NPs to practice within their full scope is inversely related to the positive effect on hospitalizations and state health outcomes
Segal	To examine potentially preventable hospitalizations rates by setting, state, medical condition, and cost	Hospital, Inpatient rehab, SNF, LTC, home health and community	Retrospective	Potentially Avoidable Hospitalization Algorithm, ICD-9 diagnosis	The national rate among MMEs for potentially avoidable hospitalizations was 133 per 1000 person-years, but there was considerable variation across health care settings. For those MMEs receiving care in a skilled nursing facility, the potentially avoidable hospitalizations rate was 690 per 1000 person-years. Five conditions were responsible for nearly 80 percent of potentially avoidable hospitalizations occurring among the study population: congestive heart failure (21%), COPD/asthma (20%), urinary tract infections (15%), pneumonia (13%), and dehydration (11%)
Anderson and Ferguson	Reduce hospital readmissions by an NP completing a systematic medication reconciliation process for all new admissions	90-bed skilled nursing facility	Pre- and postimplementation design	Evidence-based workflow process, chi-square analysis	19.2% hospital readmission rate pre implementation and 13.5% postimplementation, a 29.7% decrease in hospitalizations within a 30-day period
Ingber	Improve the overall health and health care of participating long-stay residents of nursing facilities, reducing potentially avoidable hospitalizations, improving quality of care, and decreasing health care spending	Long-stay residents—total of 143 nursing facilities	Mixed methods, quantitative and qualitative analysis, multivariate regression	Analysis of claims and assessments, site visits, interviews, and surveys	Multipronged NP interventions reduce potentially avoidable hospitalizations and related Medicare expenditures
Kane	Assess the quality of care using Evercare approach which employs NPs to provide additional primary care over and above that provided by physicians	Nursing home residents	Experimental	Data from MDS, Medicare, and United Healthcare, survival analysis, risk adjustment methods applied to the quality indicators	Hazard rates significantly lower for Evercare residents, residents had fewer preventable hospitalizations
Krichbum	Test effectiveness of nursing intervention model to improve health function, and return-home outcomes using a gerontological advanced practice nurse (GAPN) to provide interventions 6 months post-acute care, making biweekly visits and/or phone calls	33 elders 65 years and older with a hip fracture	Randomized clinical trial	Mini Mental Status Exam (MMSE), Global (GH) self-ratings, Geriatric Depression Scale (GDS), Functional Status Index (FSI) and listing of living situation	Improved function in mobility, home chores, and personal care activities
Miller	Evaluate how receipt and timing of nursing home palliative care consultations by nurse practitioners with palliative care expertise are associated with end-of-life care transitions and acute care use	Nursing home residents who died from 2006 to 2010 living in 46 nursing homes in two states	Propensity score-matched retrospective cohort study	Multivariate logistic regression analysis	Lower rates of hospitalizations, improved end-of-life nursing home care
Ploeg	Report the perceptions of residents and family members about the role of the nurse practitioner in long term care settings	35 residents and family members from four long-term care settings that employed a nurse practitioner	Qualitative descriptive approach, individual and focus groups interviews	Conventional content analysis was used to identify themes and subthemes	Perceptions of residents and family members of the nurse practitioner role in long-term care setting consistent with person-centered and relationship-centered care, enhanced quality of care, positive care experience
Poghosyan	Determine the impact of state and organizations on nurse practitioner practice environment	291 nurse practitioners in MA and 278 nurse practitioners in NY	Cross-sectional survey design; online surveys	Nurse Practitioner Primary Care Organizational Climate Questionnaire	NPs have positive perceptions of their relationship with physicians, NP’s perceptions of the relationships they have with administration is deficient, state and organizational policies should be taken into consideration to ensure least restrictive practice
Rantz	Review impact of advance practice registered nurses (APRNs) on the quality measure scores of 16 nursing homes participating the Missouri Quality Initiative (MOQI) intervention	16 nursing homes	Two-group comparisons	Data was collected for 36 months and analysis of results conducted to create a composite quality measure score for each facility, Interventions to Reduce Acute Care Transfers (INTERACT) using the Stop and Watch and Situation, Background, Assessment, Recommendation (SBAR)	APRNs working full-time in nursing homes positively influenced quality of care, reduced unnecessary hospitalizations and emergency room transfers, improved the process of transitioning between inpatient hospitals and nursing facilities, and reduced overall healthcare spending without restricting access to care
Rosenfeld	Determine the national practice patterns of nurse practitioners providing care in long-term care facilities	All physicians who are members of the American Medical Directors Association (AMDA)	Mailed survey	The survey mailed out included six domains: (1) the number of LTC facilities that have NPs involved in providing care; (2) the number of NPs engaged in care at these facilities; (3) the types of employment/financial arrangements between NPs and LTC facilities; (4) the types of services provided by the NPs; (5) the effectiveness of the NPs as perceived by the medical director; and (6) the perceived future demand for NPs in LTC	NPs involved in LTC are more likely to be involved in facilities with 100+ beds, performed a wide range of services, are effective at maintaining physician, resident, and family satisfaction, were highly effective with regard to emergency room transfers, increasing the quality of care, survey preparedness, and hospital admissions. Respondents to the survey were overwhelmingly positive about working with NPs
Ryskina	Describe current practice behaviors to identify areas where interventions could improve post-acute care outcomes	Fee-for-service Medicare beneficiaries, 65+ years old, discharged from an acute care hospital to a SNF in the period of January 2012–October 2014	Retrospective	Aggregate data from Medicare claims January 2012–October 2014, supplemental information from MDS and Provider of Services	Timely access to physicians or NPs after hospital discharges to a SNF depends on local practice patterns, not clinical needs
Kaasalainen	To evaluate the level of involvement of nurse practitioners (NPs) in activities related to preventing and managing fractures in long-term care (LTC)	Long term care (LTC)	A cross-sectional survey, qualitative interviews	The first section focused on demographic information, the second section gathered information on practice patterns of NPs related to fracture-risk assessment, post-fracture management and use of evidence-based guidelines, the third section surveyed on the processes NPs would follow to respond to related care decisions, and last section focused on identification of the barriers and facilitators faced by NPs to prevent and manage fractures	The finding suggest that the NP were involved in caring for residents’ post fractures and in risk factors assessments. The role of NP in managing fractures can be optimized by addressing barriers such as inadequate staffing at the facility and lack of timely access to diagnostic services
Ouslander, Naharci	To determine if conducting root cause analyses (RCA) on transition of residents from skilled nursing facility to hospitals can help prevent preventable emergency department (ED) visits and hospitalizations	Sixty-four of 88 SNFs	Retrospective, quasi-experimental	INTERACT (Interventions to Reduce Acute Care Transfers), Quality Improvement (QI) tool	The studies indicate that using RCAs provides important insights to factors contributing to the transfers, propose several areas of attention for process improvements and related education which may help reduce preventable hospitalizations
Kuo	The use of nurse practitioners (NPs) is one way to address the shortage of physician primary care providers	Medicare beneficiaries aged 65 or older with Parts A and B coverage and not in a health maintenance organization (HMO) for the entire twelve months of each year during 1998–2010	We identified individual providers by their Unique Provider Identification Number for 1998–2007 and National Provider Identifier for 2007–10	Hierarchical generalized linear mixed models	The overall number of NPs reimbursed for evaluation and management services in the 5 percent Medicare claims data rose from 3114 in 1998 to 37,638 in 2010
Mullaney	To understand the impact of mortality risk assessments (MRAs) and advance care planning (ACP) discussions completed by nurse practitioners (NP) on clinical outcomes for newly registered Medicare Advantage nursing home residents	The final sample of 87 patients was 72% female with a mean age of 81 years, LTC	Prospective, nonexperimental approach	Mortality Risk Assessment (MRA) & Advance Care Planning (ACP)	The study demonstrated positive clinical outcomes post ACP discussions. The outcomes include increase in number of patients with a comfort goal of care, decline in full-code status patients, reduced hospitalizations and improved quality in end-of-life care
Cole	NPs collaborative practice with physician and nursing colleagues to reduce hospitalization	190-bed residential care facility in New Brunswick, LTC	Retrospective	Daily clinical monitoring	As shown in this case, the presence of an NP clearly impacted a reduction in emergency room visits and hospitalizations, events that often accelerate further physical, mental, and functional decline particularly among the frail elderly
Hullick	To examines the impact of the aged care emergency services (ACE) on residential aged care facilities (RACF) residents’ transfers to hospitals	Four RACFs, LTC	Prospective, retrospective	Generalized estimating equations	This study has demonstrated that a complex multi-strategy intervention led by nursing staff can successfully reduce hospital admissions for older people living in Residential Aged Care Facilities
Ordonez	To examine the outcome of a gerontological nurse practitioner (GNP) care coordination model on healthy transition and 30-day rehospitalizations among older adults. In this study, the patients are discharged from a hospital to a SNF for rehabilitation post coronary artery bypass graft (CABG) surgery	10 older adults with age > 65 years status post CABG. Skilled nursing facility	Retrospective, quasi-experimental	Scale 1-4 including control of signs and symptoms, functional status, depression, sense of integrity	Findings indicate five to eight percent more effectiveness in outcomes with GNP care coordination model than the standard of care. The project demonstrates the effectiveness of the GNP care coordination model in decreasing the risk of 30-day rehospitalization and facilitating the development of realistic and achievable goals
Dwyer	Reducing avoidable hospitalizations of aged care facility (ACF) residents can improve the resident experience and their health outcomes	Aged care facility (ACF), LTC	Retrospective	Donabedian model	The studies indicate that the NPs with their advanced clinical skills and prescribing rights, were able to deliver a range of timely health services within the ACF in the absence of a PCP. This resulted in reduced hospitalization and managing of care of residents at the facility
Reidt	To study an interprofessional collaborative practice model aimed to improve discharge management from the transitional care unit of the skilled nursing facility (SNF) to home. The model includes a geriatrician, nurse practitioner and a pharmacist	SNF, LTC	Prospective, Retrospective	Comparison of intervention and control groups	This study suggests that collaboration among a geriatrician, nurse practitioner, and pharmacist may be an effective means of decreasing hospitalizations and ED visits within 30 days after SNF discharge
Arendts	To enhance quality of life and reducing hospitalizations for people living in residential aged care facilities (RACF)	Six facilities (352 beds each) were included, RACF, LTC	Prospective, Retrospective, quasi-experimental. A cluster controlled clinical trial of nurse practitioner care in RACF. Six facilities were included: three randomly allocated to intervention where nurse practitioners working with general practitioners and using a best practice guide were responsible for care, and three control	Modified Barthel Index (MBI), Psychogeriatric Assessment Scale (PAS) assessments	Nurse practitioner care coordination resulted in no statistically significant change in rates of ED transfer or health care utilization, but better maintained resident quality of life

**Table 2:** Matrices Showing Theme Occurrence.

Facilitators	Occurrences	Sum	%
Decreased hospitalizations	7,8,9,10,11*,12*,13,14*,15*,16,17*,18,19,20*,21,22,23*,24,25,26,27*	37	27.21%
Improved health outcomes	7,9*,12,14,20*,21*,23*,24*,26,28*,29*,30,31	27	19.85%
Improved quality of care	8*,13,14*,18,19,20*,23*,24,26,27,28*,31,33	26	19.12%
Increased access to healthcare	18,20,23,24*,25,30,31*,33,34*	14	10.29%
Unrestrictive or least restrictive scope of practice for NPs	10,14,16,17,31,32,33,34,35,36*	10	7.35%
Reduced healthcare costs	7,8,9*,12,14,23,30	9	6.62%
Multidisciplinary approach	21*,25,29,32	5	3.68%
Improved end of life care	16*	3	2.21%
Patient and family education	13,20*	3	2.21%
Improved patient and family satisfaction	19,32	2	1.47%
		136	
**Barriers**	**Occurrences**	**Sum**	**%**
Restrictive scope of practice for NPs	9,14,16,20,23,24,31*,32*,34,35,36	13	28.89%
Poor quality of care	13*,19,20,22,27,28*	11	24.44%
Lack of access to healthcare	17,20,22*,27,29	7	15.56%
Inadequate staffing	8,10,17,23,28,33	6	13.33%
Decreased patient satisfaction	17,23,29	3	6.67%
Poor decision making	18*	2	4.44%
Increased hospitalizations	19	1	2.22%
Increased healthcare costs	19	1	2.22%
Poor communication	36	1	2.22%
		45	
